# Healthcare services for low-wage migrant workers: A systematic review

**DOI:** 10.1016/j.socscimed.2025.118176

**Published:** 2025-09

**Authors:** Eilin Rast, Karen Lau, Rosita Chia-Yin Lin, Tharani Loganathan, Sally Hargreaves, Cathy Zimmerman, on behalf of the Consortium for Migrant Worker Health

**Affiliations:** a London School of Hygiene and Tropical Medicine, London, UK; b Department of Population Medicine & Health Services Research, School of Public Health, Bielefeld University, Bielefeld, Germany; c The Migrant Health Research Group, Institute for Infection and Immunity, City St George's, University of London, London, UK; d Centre for Epidemiology and Evidence-Based Practice, Department of Social and Preventive Medicine, Faculty of Medicine, University of Malaya, Kuala Lumpur, Malaysia; e Department of Global Health & Development, London School of Hygiene & Tropical Medicine, London, UK

## Abstract

Low-wage labour migrants often face health-damaging living and working conditions, but are frequently excluded from healthcare. The othering of migrants, bordering of healthcare and simple oversight and negligence create widening health inequalities for a society's essential workers. This review aimed to identify the forms and effectiveness of healthcare services designed to make healthcare accessible for migrant workers.

We searched for literature through Medline, Embase, Global Health, Web of Science, and Global Index Medicus (from 1 January 2000 till 9 June 2023), focussing on selected work sectors (domestic work, construction, manufacturing, agriculture, mining). Primary research, reports, and grey literature from 2000 onwards containing descriptions or evaluations of healthcare services exclusively targeting low-wage migrant workers and their families were included. We excluded services focussing only on specific health conditions or disease screening. Quality appraisal was based on tools from the Joanna Briggs Institute. We narratively synthesised service characteristics and effects. This review follows the PRISMA reporting guidelines for systematic reviews and is registered with PROSPERO (CRD42023459360).

Identified studies included 21 healthcare services targeting low-wage migrant workers in six countries (China, Dominican Republic, Italy, Qatar, South Africa, USA) in three sectors (agriculture, manufacturing, domestic work). Services included established medical facilities (e.g., general hospital care, semi-permanent primary healthcare (PHC) services); mobile clinics for PHC; and telehealth services. The healthcare services were provided by governmental, non-governmental, academic, and private actors. Most targeted migrant farmworkers and were primarily located in the United States. Common healthcare barriers were addressed, for example, via free care, outreach, or non-traditional hours. However, service effects on health, access and uptake, patient satisfaction, and acceptability were largely unclear, as only six studies offered some fragmentary evaluative evidence.

Few healthcare services targeting migrant workers have been documented and evaluated, especially in LMICs. Although migrant workers are deemed to be mobile populations, once in the destination location, many are quite immobile when it comes to accessing healthcare. Thus, in the face of persistent exclusion of migrant workers, health systems cannot simply rely on the ability of this vital workforce to seek and use preventative or curative care, but healthcare services must be actively designed to be accessible to this mobile population in order to ensure health as a human right.

## Highlights

• Low-wage migrant workers face particular health risks and barriers to healthcare.
• We reviewed targeted stationary and mobile clinics and telehealth globally.
• The identified services offer valuable examples to overcome healthcare barriers.
• However, few targeted healthcare services have been documented and evaluated.
• Healthcare systems need to be made more migrant-sensitive to avoid exclusion.

## 1 Introduction

Labour migrants make important contributions to the global economy (International Labour Organization, 2021; World Bank Group, 2024). According to conservative estimates, the number of international labour migrants has been steadily increasing, reaching 169 million in 2019 (International Labour Organization, 2021), with greater estimates of internal labour migration (McAuliffe and Triandafyllidou, 2021). Although many mobile workers are in labour arrangements that generally benefit their income, many are engaged in low-wage jobs associated with health risks (Abubakar et al., 2018; International Labour Organization, 2010; International Labour Organization et al., 2022; Mucci et al., 2019). Moreover, low-wage work in general is often precarious, i.e., dominated by insecurity, informality, and limited workers' rights (Aktas et al., 2022; European Observatory of Working Life, 2018; Grimshaw, 2011). Given the multiple disadvantages related to migrant status, especially for irregular international migrants (e.g., possible language barriers, limited social support networks, lack of labour and social protection, poor housing options), low-wage migrant workers are often more vulnerable to exploitation than non-migrant workers and have an increased risk of being trafficked for labour (Abubakar et al., 2018; Hargreaves et al., 2019; International Labour Organization et al., 2022). Furthermore, labour migrants are often employed in sectors which are known for exploitative and forced labour conditions (International Labour Organization, 2021; International Labour Organization et al., 2022). Considering labour arrangements to span a wide spectrum between ‘decent’ (International Labour Organization, n.d.) and ‘forced’ work, migrant workers thus face particular structural disadvantages that make them more prone to experience working conditions that are located more towards the forced labour end.

Simultaneously, evidence on the social gradient in health (Marmot, 2016; World Health Organization, 2008) indicates that low-wage work, which is commonly occupied by labour migrants, is associated with poor health outcomes, both directly through harmful work conditions and indirectly because of socioeconomic disadvantages (Baron et al., 2014; Flynn, 2021; Ingram et al., 2021). Although working conditions may vary geographically and by labour activity, high levels of occupational hazards (e.g., exposure to toxins, frequent accidents, repetitive movements, and extreme temperatures), extensive working hours, insecure employment, and substandard living conditions (including overcrowding and financial insecurity) are widespread (Baron et al., 2014; Buller et al., 2015; Goldman et al., 2021; Gottlieb et al., 2025; Ingram et al., 2021; Pocock et al., 2018; Saldaña-Villanueva et al., 2023; Stiehl et al., 2018). Indeed, these work conditions are often crudely described as the 3Ds: ‘Dirty’, ‘Difficult’ and ‘Dangerous’. These unhealthy conditions for migrant workers can easily be associated with othering: Social categorisation processes that manifest in social structures, institutions, discourses and language that promote and reinforce group-based inequalities, also faced by other migrant groups (Akbulut and Razum, 2022; Grove and Zwi, 2006; Ladegaard, 2022). With a particular emphasis on power asymmetries, othering as an analytical lens points to the intersectionality of different social categories (Akbulut and Razum, 2022) – such as low socioeconomic status or migrant and ethnic minority status in the case of low-wage labour migrants – and their exclusionary, disempowering and marginalising effects (Akbulut and Razum, 2022; Grove and Zwi, 2006), which manifest in racism and other forms of social exclusion of labour migrants (Baron et al., 2014; Grimshaw, 2011; Krieger, 2010; Stiehl et al., 2018). Consequently, multiple poor health outcomes are associated with the work commonly performed by labour migrants, including conditions that affect their physical (e.g., respiratory, musculoskeletal, dermatological, and infectious diseases, injuries), mental and social health (e.g., violence, substance addiction, isolation, common mental disorders) (Abubakar et al., 2018; Aktas et al., 2022; Arcury and Quandt, 2007; Baron et al., 2014; Buller et al., 2015; Goldman et al., 2021; Ingram et al., 2021; Mucci et al., 2019; Ottisova et al., 2016; Pocock et al., 2018; Saldaña-Villanueva et al., 2023; Stiehl et al., 2018; Zimmerman and Kiss, 2017). In a meta-analysis of data on 7,260 labour migrants, almost half had at least one occupation-related morbidity (Hargreaves et al., 2019). In addition to general healthcare needs, low-wage migrant workers may face specific or greater health and occupational safety needs that require medical attention than individuals with safer jobs and more health-promoting living and working conditions. Yet, despite their exposure to riskier working and living conditions that may require healthcare, studies repeatedly indicate that low-wage migrant workers often have difficulty accessing healthcare (Aktas et al., 2022; Buller et al., 2015; Luo and Escalante, 2018; Pega et al., 2021; Stiehl et al., 2018).

At the same time as states depend on migrant labour, contemporary health systems generally maintain systemic bordering practices. Bordering is the dislocation of state borders from their territorial limits, making borders penetrate state institutions and thus everyday life, while determining belonging and non-belonging (Yuval-Daṿis et al., 2019). Bordering practices render health systems agnostic, negligent or, at worst, hostile to mobile populations. That is, health systems are often exclusionary, maintaining institutional bordering that intentionally or inadvertently separates wanted and unwanted service recipients (Akbulut and Razum, 2022; O'Donnell et al., 2018). Scholars have noted that many health systems are based on othering as a multidimensional social phenomenon, which helps explain the links between minority status and health inequalities (Akbulut and Razum, 2022). Authors have also highlighted how ‘securitisation’ has served as a vehicle that operationalises power structures (e.g., nationalism, race, gender, class) that may be driven by health concerns and yet negatively affect health access (Innes, 2024). Security structures can set the boundaries that create contested identities, and divisions of who belongs and who is overlooked or actively banished (Innes, 2024; Loganathan et al., 2024). Migrant workers are emblematic of those who are often among those least able to access traditional or mainstream service models (e.g., site-based clinics; health promotion in local languages) (Abubakar et al., 2018; Arcury and Quandt, 2007; Loganathan et al., 2019; Santalahti et al., 2020; Simon et al., 2015; World Health Organization, 2022), due to which they have to rely on services that overcome common access barriers.

Healthcare access has been defined as “the opportunity to have health care needs fulfilled” (Levesque et al., 2013). Levesque et al., propose five access dimensions: approachability, acceptability, availability, affordability and appropriateness of services, which are associated with provider and patient characteristics (Levesque et al., 2013). Many of these features can be found in structural and individual bordering of healthcare access, including questions of ‘us’ and ‘them’ and ‘self’ and ‘other’ identities (Cassidy and Davidson, 2024; Vollmer, 2021). Drawing on the five access dimensions, we developed a conceptual framework for this review, which applied commonly reported access barriers (Santalahti et al., 2020; Simon et al., 2015; World Health Organization, 2022) (Supplement 1). Constraints that often impact populations at large include direct and indirect costs, inadequate insurance coverage, geographical distance, lack of affordable transport, work-related time constraints, and service gaps (Baron et al., 2014; Buller et al., 2015, Ingram et al., 2021; Simon et al., 2015). Migrant workers often encounter further access barriers related to their legal status and missing documents (e.g., passport and work permits), language and cultural differences, mobility that hinders the continuity of care, discrimination by health system representatives, and challenges due to being unfamiliar with local care structures and entitlements (Abubakar et al., 2018; Arcury and Quandt, 2007; Loganathan et al., 2019; Santalahti et al., 2020; Simon et al., 2015; World Health Organization, 2022). For example, even where documented migrant workers are covered by mandatory healthcare insurance schemes, it is not uncommon for workers to be unaware of their entitlements to care and for medical fees to be higher than for citizens (Loganathan et al., 2020). Inequitable healthcare access has been conceptualised as determined by social characteristics and access-enabling resources (e.g., insurance, time, and service availability) rather than need (Andersen, 1995). To achieve universal health coverage as envisioned by the United Nations' Sustainable Development Goals' target 3.8 (United Nations, 2016), and to realise the right to health as a human right (Abubakar et al., 2018; Office of United Nations High Commissioner for Human Rights & World Health Organization, 2008), health systems need to adapt to the lived realities of low-wage labour migrants, which influence their health needs and form the context of healthcare seeking (Supplement 1).

Several relevant literature reviews have been conducted over the past 20 years, specifically on healthcare services for migrant farmworkers in the USA (Arcury and Quandt, 2007; Bloss et al., 2022; Luque and Castañeda, 2013; Villarejo, 2003). Furthermore, evidence on workplace health promotion programmes for migrant workers across the globe has been compiled, but without including medical services (Evagora-Campbell et al., 2022). Therefore, despite the need to improve healthcare access for low-wage migrant workers (Buller et al., 2015; Hargreaves et al., 2019), knowledge on existing healthcare services specifically targeting this population and the effects of these services remains limited, impeding evidence-informed policies and interventions (Abubakar et al., 2018; Aktas et al., 2022; Arcury and Quandt, 2007; Luque and Castañeda, 2013).

To fill this knowledge gap, we reviewed healthcare services that specifically and exclusively targeted migrant workers in sectors associated with low-wage and forced labour. The following questions guided our review:

1) What are the characteristics of healthcare services that specifically target low-wage migrant workers?
2) How do these healthcare services influence healthcare access and health-related outcomes (including physical and mental health and well-being, service access and uptake, patient satisfaction and acceptability, and cost-effectiveness) (Supplement 1)?

## 2 Methods

We conducted a systematic review following PRISMA guidelines (Page et al., 2021) (see Supplement 2 for PRISMA Checklist) and registered a protocol (PROSPERO: CRD42023459360) (Rast et al., 2023), from which we deviated by narrowing the review's focus down from low-wage workers in general to low-wage migrant workers.

### 2.1 Inclusion and exclusion criteria

We included qualitative and quantitative primary studies and reports (published in English or French from 2000 onwards) containing descriptions or evaluations of healthcare services exclusively targeting low-wage migrant workers and their families. Full texts needed to detail at least the target population, services provided, and staff composition for inclusion. We aimed to identify examples that enable migrant workers (and their families) to receive a range of general healthcare services (e.g., general primary medicine, maternal health, dental care, mental health, occupational health services) provided by mobile clinics, clinics on worksites, or other established (or place-based) clinics. To consider a certain level and immediateness of carethat also allows for curative elements, only services provided by healthcare professionals (e.g., physicians, nurses, psychiatrists, midwives) were eligible. In addition, we included telehealth services to examine approaches for overcoming different access barriers and assuring continuity of care for mobile populations (Marcin et al., 2016; Truong et al., 2022). The population of interest is internal and international migrant workers worldwide who are likely to receive low pay under exploitative or otherwise precarious working conditions. We therefore focussed on sectors commonly associated with exploitative work (domestic work, construction, manufacturing, agriculture/forestry/fishing, mining) by drawing on the International Labour Organisation's 2016; 2021 Global Estimates of Modern Slavery (International Labour Office, 2017; International Labour Organization et al., 2022). By drawing on these global estimates we capitalised on the best available evidence regarding the role of exploitative work in different labour sectors, but do not claim that migrant workers as a group should be equated with exploited or forced labourers. Rather, choosing these sectors is an attempt to identify work sectors particularly affected by precarious and exploitative working conditions, without wanting to determine whether empirical examples from the reviewed literature meet the definition of, e.g., ‘forced labour’.

We excluded unclear or mixed-income groups, non-migrants, and commercial sex workers (given the comparatively more research on this sector (Abad et al., 2015; Buller et al., 2015; Dhana et al., 2014; Jeal et al., 2015; Johnson et al., 2023; Rinaldi et al., 2018; Turner et al., 2022)) as well as services focussing only on specific diseases, vaccination, screening, and emergency care, interventions to increase access to the wider health system (e.g. information campaigns, health insurance schemes), services also targeting other patient groups, and health promotion interventions, which have been reviewed elsewhere (Evagora-Campbell et al., 2022; Pham et al., 2020; Stiehl et al., 2018). Inclusion and exclusion criteria were developed within the PICO framework (McKenzie et al., 2023) (see Supplement 3).

### 2.2 Search strategy

**Fig. 1:**
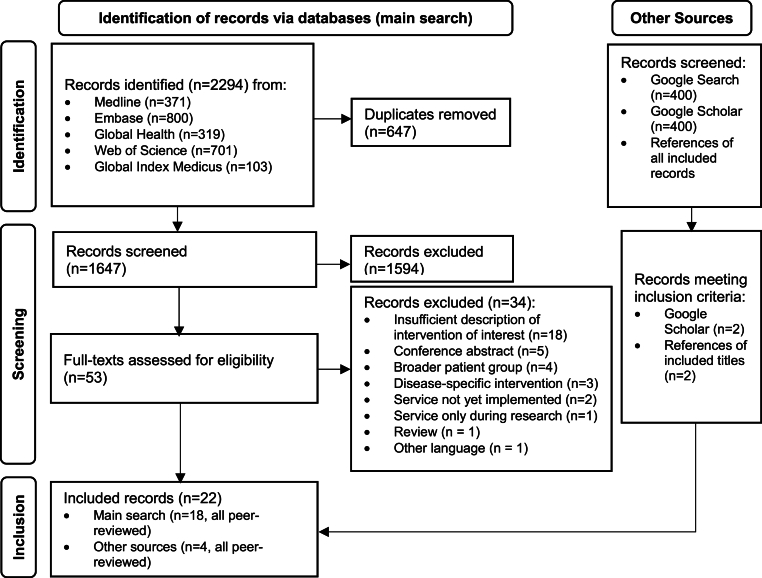
PRISMA flow diagram presenting the selection of references.

We searched Medline, Embase, Web of Science, Global Health, and Global Index Medicus for studies and reports on 9 June 2023 by combining free-text terms and subject headings related to the healthcare services AND work conditions AND work sectors of interest (see Supplement 4). To identify further published and grey literature, we simplified the search strategy for searches in Google Search and Google Scholar and hand-searched the bibliographies of all included references. Records were deduplicated (Falconer, 2018) and uploaded into Rayyan (Ouzzani et al., 2016) for duplicate screening. Titles or abstracts had to mention health services for further inclusion. During full text screening, we documented the primary reason for exclusion (Fig. 1).

### 2.3 Critical appraisal

The quality of those studies examining service effects was critically appraised, independently by two reviewers, using JBI Critical Appraisal Tools (Joanna Briggs Institute), with scores encompassing low, medium and high study quality. For mixed-methods studies, we appraised the study component (i.e., qualitative or quantitative) reporting relevant outcomes. Discordant appraisals were discussed until an agreement was reached. Quality did not determine inclusion but was considered in the analysis.

### 2.4 Data extraction

Using a customised form, we extracted general information on the study or report, service characteristics, patient population, context, as well as challenges and facilitators of the service. Furthermore, we collected information on how services influenced healthcare access within the framework by Levesque et al. (2013). For research studies examining service effects (on physical and mental health outcomes, patient satisfaction and acceptability, healthcare access and uptake, or cost-effectiveness), we furthermore collected information on the relevant outcomes. The first author extracted the data with verification by the second and third authors.

### 2.5 Data synthesis and analysis

Included healthcare services were tabulated and ordered by the primary mode of service delivery (i.e., established, mobile, or telehealth service) for sub-group analysis. The first part of the synthesis encompasses all included titles, summarising service characteristics and the impact on healthcare access by drawing on the framework by Levesque et al. (2013). The second part, limited to a subset of studies, narratively synthesises service effects.

## 3 Results

### 3.1 Characteristics of included studies and reports

Of 2294 records from the databases and further references from other sources (including search of grey literature) we included 22 titles from the academic literature (Brumitt et al., 2011; Burgel et al., 2004; Chen et al., 2010; Connor et al., 2007, 2010; Corwin et al., 2021; Crouse et al., 2010; Di Gennaro et al., 2021; Etienne et al., 2016; Garcia et al., 2012; Gruchy and Kapilashrami, 2019; Heravi and Bertram, 2007; Ingram et al., 2015; Latoo et al., 2021; Lausch et al., 2003; Hiebert and Vargas, 2015; Liem et al., 2022; Lukes and Simon, 2006; Luque et al., 2012; Parikh et al., 2010; Price et al., 2013; Qian et al., 2007). Most of them were descriptive reports (Brumitt et al., 2011; Connor et al., 2007, 2010; Corwin et al., 2021; Garcia et al., 2012; Heravi and Bertram, 2007; Latoo et al., 2021) (two relating to the same healthcare service (Connor et al., 2010; Connor et al., 2007)) or studies not focussing on service effects (Burgel et al., 2004; Di Gennaro et al., 2021; Etienne et al., 2016; Gruchy and Kapilashrami, 2019; Ingram et al., 2015; Lausch et al., 2003; Luque et al., 2012; Parikh et al., 2010; Qian et al., 2007). Only six studies (Chen et al., 2010; Crouse et al., 2010; Liem et al., 2022; Lukes and Simon, 2006; Hiebert and Vargas, 2015; Price et al., 2013) examined relevant service effects, but were of mixed quality.

### 3.2 Characteristics of healthcare services

Most of the 21 different healthcare services (see overview table in Supplement 5) were implemented in the USA (Brumitt et al., 2011; Burgel et al., 2004; Connor et al., 2007, 2010; Corwin et al., 2021; Garcia et al., 2012; Heravi and Bertram, 2007; Ingram et al., 2015; Lausch et al., 2003; Lukes and Simon, 2006; Luque et al., 2012; Price et al., 2013), followed by the Dominican Republic (Crouse et al., 2010; Etienne et al., 2016; Hiebert and Vargas, 2015; Parikh et al., 2010), China (Chen et al., 2010; Liem et al., 2022; Qian et al., 2007), Italy (Di Gennaro et al., 2021), Qatar (Latoo et al., 2021), and South Africa (Gruchy and Kapilashrami, 2019). Except for two Chinese healthcare services for internal migrant workers (Chen et al., 2010; Qian et al., 2007), services targeted international migrant workers and their families.

Services consisting of established (or place-based) health facilities (Burgel et al., 2004; Chen et al., 2010; Garcia et al., 2012; Heravi and Bertram, 2007; Ingram et al., 2015; Latoo et al., 2021; Lausch et al., 2003; Lukes and Simon, 2006; Qian et al., 2007) and mobile clinics (Brumitt et al., 2011; Connor et al., 2007, 2010; Corwin et al., 2021; Crouse et al., 2010; Di Gennaro et al., 2021; Etienne et al., 2016; Gruchy and Kapilashrami, 2019; Hiebert and Vargas, 2015; Luque et al., 2012; Parikh et al., 2010) were described by nine and 11 titles respectively. Another two studies reported on telehealth apps (Liem et al., 2022; Price et al., 2013). Some healthcare services also combined place-based, outreach, and telehealth (Corwin et al., 2021; Latoo et al., 2021; Lausch et al., 2003). While most titles reported on individual local services, a few focussed on the US-wide system of migrant health centres (Garcia et al., 2012; Lukes and Simon, 2006) or the subnational occupational health system in the Chinese district Bao'an (Chen et al., 2010).

Stationary healthcare facilities were of heterogeneous scales and scope, ranging from a general hospital in an industrial area in Qatar (Latoo et al., 2021) to primary healthcare provided on weekends in established medical centres in the USA (Heravi and Bertram, 2007). Most of these facilities offered primary or occupational health services. The occupational health system in Bao'an was established to complement primary healthcare structures (Chen et al., 2010). Mobile clinics offered primary healthcare, sometimes also including more specialised services, such as dental, maternal, and paediatric care or physiotherapy (Connor et al., 2007, 2010; Crouse et al., 2010; Di Gennaro et al., 2021; Etienne et al., 2016; Gruchy and Kapilashrami, 2019; Parikh et al., 2010). The two telehealth interventions were apps for mental health (Liem et al., 2022) and chronic disease management (Price et al., 2013). Health education and other health promotion commonly formed part of the healthcare services (e.g., stretching (Brumitt et al., 2011; Burgel et al., 2004), occupational health and safety measures (Burgel et al., 2004), or patient support groups (Gruchy and Kapilashrami, 2019)). A few programmes also addressed wider social determinants of health through food supplementation (Parikh et al., 2010), donated goods (Etienne et al., 2016), or comprehensive social services (Corwin et al., 2021). Healthcare staffing in established facilities ranged from big interdisciplinary and highly specialised teams (Latoo et al., 2021) to nurse-led satellite clinics (Lausch et al., 2003). Mobile clinics were operated by smaller teams of nurses and physicians or by nurses alone (Gruchy and Kapilashrami, 2019), but medical specialties were rarely detailed. Some services were supported by additional voluntary health professionals, including healthcare students (Brumitt et al., 2011; Burgel et al., 2004; Connor et al., 2007, 2010; Luque et al., 2012), and two were exclusively volunteer-run (Etienne et al., 2016; Heravi and Bertram, 2007).

Agricultural workers dominated as a target group (16 out of 21 interventions), including all but one healthcare service in the USA, all in the Dominican Republic, and all mobile clinics (Brumitt et al., 2011; Connor et al., 2007, 2010; Corwin et al., 2021; Crouse et al., 2010; Di Gennaro et al., 2021; Etienne et al., 2016; Garcia et al., 2012; Gruchy and Kapilashrami, 2019; Heravi and Bertram, 2007; Ingram et al., 2015; Lausch et al., 2003; Lukes and Simon, 2006; Hiebert and Vargas, 2015; Luque et al., 2012; Parikh et al., 2010; Price et al., 2013). Established, non-mobile clinics were also provided in manufacturing (Burgel et al., 2004; Chen et al., 2010; Garcia et al., 2012; Latoo et al., 2021; Qian et al., 2007). The only intervention implemented among (but not exclusively) domestic workers was the mental health app (Liem et al., 2022). Construction workers were only mentioned once in the context of US migrant health centres, which mainly serve farmworkers (Garcia et al., 2012).

Actors involved in planning and implementing the healthcare services included private corporations, governmental bodies, academic institutions, and NGOs (international or local civil society organisations). While no collaboration between NGOs and private actors occurred, all other combinations and individually-led services were reported. Established clinics resulted from either governmental (Chen et al., 2010; Latoo et al., 2021) or private sector initiatives (Qian et al., 2007). The governmental occupational health system in Bao'an, e.g., involved factory employers through partial funding and occupational health training (Chen et al., 2010). Only smaller, semi-permanent services involved NGOs (Burgel et al., 2004; Heravi and Bertram, 2007), with the exception of federally-qualified health centres in the USA which count as community-based organisations (Garcia et al., 2012; Ingram et al., 2015; Lausch et al., 2003; Lukes and Simon, 2006). Mobile clinics mostly involved local or international NGOs (Crouse et al., 2010; Di Gennaro et al., 2021; Etienne et al., 2016; Hiebert and Vargas, 2015), at times with academic (Brumitt et al., 2011; Connor et al., 2007, 2010; Luque et al., 2012; Parikh et al., 2010) and governmental partnerships (Gruchy and Kapilashrami, 2019). The two telehealth interventions were developed and implemented by universities (Price et al., 2013), in one case supported by community organisations (Liem et al., 2022).

For about half of the healthcare services, the source of funding was not discernible. Based on the information available, established health facilities were mainly government-funded (Garcia et al., 2012; Ingram et al., 2015; Latoo et al., 2021; Lausch et al., 2003; Lukes and Simon, 2006), but the two Chinese industrial clinics were fully or partly paid for by the operating company (Chen et al., 2010; Qian et al., 2007). Mobile clinics were funded by governmental (Corwin et al., 2021), NGO (Di Gennaro et al., 2021; Etienne et al., 2016; Gruchy and Kapilashrami, 2019; Hiebert and Vargas, 2015), and academic actors (Connor et al., 2007, 2010; Parikh et al., 2010). Difficulties in acquiring necessary resources, including staff, clinic sites, and funding, were the most frequently mentioned challenge (Chen et al., 2010; Connor et al., 2010; Gruchy and Kapilashrami, 2019; Lausch et al., 2003; Liem et al., 2022; Lukes and Simon, 2006), while collaborations with other healthcare providers (Garcia et al., 2012; Gruchy and Kapilashrami, 2019; Latoo et al., 2021; Lausch et al., 2003), community organisations (Connor et al., 2007; Heravi and Bertram, 2007; Liem et al., 2022), and employers (Chen et al., 2010; Corwin et al., 2021) were commonly reported as facilitating the healthcare services.

### 3.3 Access to healthcare services

The reviewed services influenced healthcare access for low-wage migrant workers across Levesque et al.’s (2013) five access dimensions (Table 1).

**Table 1:** Healthcare access dimensions addressed by interventions targeting migrant workers.

Access dimension (Levesque et al. (Levesque et al., 2013))	Barriers in wider health system	Ways in which healthcare services addressed these barriers
**Approachability** • Transparency • Outreach • Information • Screening **Ability to perceive** • Health literacy • Health beliefs • Trust • Expectations	Lack of knowledge about services and eligibility	Make services known through outreach activities • with community visits (Connor et al., 2007, 2010) • by involving community support organisations and community health workers (Burgel et al., 2004; Gruchy and Kapilashrami, 2019; Heravi and Bertram, 2007; Ingram et al., 2015; Liem et al., 2022; Parikh et al., 2010) • by using media channels (radio, television, social media) (Burgel et al., 2004; Liem et al., 2022) Support in navigating services • through community health workers (Ingram et al., 2015; Parikh et al., 2010) • by designing simple care pathways (Latoo et al., 2021) • support with scheduling follow-ups (Lausch et al., 2003; Luque et al., 2012) • pointing out healthcare providers in next destination (personal/digital help) (Garcia et al., 2012; Gruchy and Kapilashrami, 2019; Price et al., 2013)
	Health literacy/health beliefs	Provide health education and health screenings (Burgel et al., 2004; Chen et al., 2010; Connor et al., 2007, 2010; Corwin et al., 2021; Etienne et al., 2016; Heravi and Bertram, 2007; Ingram et al., 2015; Lausch et al., 2003; Luque et al., 2012)
	Lack of trust, e.g. due to undocumented legal status	Generate trust through • collaborations with community support groups and community health workers (Heravi and Bertram, 2007; Ingram et al., 2015) • through long-term (Connor et al., 2010; Corwin et al., 2021) and regular engagement (Lausch et al., 2003) with the patient community
**Acceptability** • Professional values/norms • Culture • Gender **Ability to seek** • Personal/social values • Culture • Gender • Autonomy	Linguistic/cultural differences	Overcome language barriers with • multilingual healthcare staff (Corwin et al., 2021; Latoo et al., 2021; Lausch et al., 2003; Lukes and Simon, 2006) • translators and community health workers (Burgel et al., 2004; Connor et al., 2010; Ingram et al., 2015) • multilingual telehealth services (Liem et al., 2022; Price et al., 2013) Address cultural differences by • employing staff with diverse cultural backgrounds (Corwin et al., 2021; Latoo et al., 2021) and cultural mediators (Di Gennaro et al., 2021) • providing culturally-sensitive care (e.g., through family-friendly clinic spaces, incorporating patients' concepts of health into the care) (Connor et al., 2010; Lausch et al., 2003) Considering stigma of mental health by providing specialised services in polyclinic (Latoo et al., 2021)
	Limited autonomy from employer/Potential job loss	Decrease dependency on employers as a barrier for seeking care through • flexible telehealth services (Latoo et al., 2021; Liem et al., 2022) • cooperations with employers (Brumitt et al., 2011; Chen et al., 2010; Connor et al., 2010; Corwin et al., 2021)

**Availability and accommodation** (physical and timely reachability) • Geographic location • Accommodation • Opening hours • Appointment mechanisms **Ability to reach** • Living environments • Transport • Mobility • Social support	Service gaps (on local or national level)	Increase service coverage through • Providing services (Gruchy and Kapilashrami, 2019; Hiebert and Vargas, 2015) or extending existing ones during peak times (Connor et al., 2010) • employment of community health workers between clinic visits (Gruchy and Kapilashrami, 2019)
	Distance, lack of transportation	Overcome transport-related barriers through • the service provision at migrant workers' residency/workplace • mobile and telehealth services
	Time	Harmonise service times with the patients' working hours by • providing services during non-traditional hours (Burgel et al., 2004; Connor et al., 2010; Corwin et al., 2021; Lukes and Simon, 2006; Luque et al., 2012) • during working hours in collaboration with employers (Brumitt et al., 2011) Reduce the time needed for health seeking though • telehealth services (Corwin et al., 2021; Latoo et al., 2021; Liem et al., 2022; Price et al., 2013) • proximity of services

**Affordability** • Direct and opportunity costs **Ability to pay** • Income, assets • Social capital • Insurance	Direct and indirect costs as well as lack of health insurance	Offer free or low-cost services and needed medical supplies, independent of insurance status (exemplified by all interventions)
	Lost wages	Harmonise opening times with working hours of the patients (Burgel et al., 2004; Connor et al., 2010; Corwin et al., 2021; Lukes and Simon, 2006; Luque et al., 2012)

**Appropriateness** (fit between services and client needs, timeliness) • Technical and interpersonal quality • Adequacy • Coordination and continuity **Ability to engage** • Empowerment • Information • Adherence • Caregiver support	Inadequate fit between services and needs	Provide services that meet migrant workers' needs • with qualified, multidisciplinary healthcare staff trained in needed specialities, such as occupational and mental health (Burgel et al., 2004; Chen et al., 2010; Garcia et al., 2012; Heravi and Bertram, 2007; Latoo et al., 2021) • by taking into account living and working conditions in the care provision (Burgel et al., 2004; Connor et al., 2010) • a referral system for more specialised or higher-level care (Brumitt et al., 2011; Burgel et al., 2004; Lausch et al., 2003; Luque et al., 2012) • with timely access to care (Latoo et al., 2021)
	Linguistic and cultural differences	Address language and cultural barriers to assure adequate patient-provider communication and the involvement of patient/community representatives (see *acceptability* above)
	Mobility	Assure continuity of care for mobile workers through • medical records transfer, transfer letters, or patient-held records (Garcia et al., 2012; Gruchy and Kapilashrami, 2019) • handing out higher supplies of medication (Gruchy and Kapilashrami, 2019) • patient navigation and referral in next destination (Garcia et al., 2012; Gruchy and Kapilashrami, 2019; Price et al., 2013) Provide telehealth services (Liem et al., 2022; Price et al., 2013)

To make services known, and thus *approachable*, among target groups, media and personal outreach as well as health education activities and were adopted (Burgel et al., 2004; Connor et al., 2007, 2010; Liem et al., 2022). The involvement of community health workers (CHWs) (Gruchy and Kapilashrami, 2019; Heravi and Bertram, 2007; Ingram et al., 2015; Parikh et al., 2010), long-term community engagement, which in some cases also entailed educational and health and safety-related activities with workers as well as employers over decades (Connor et al., 2010; Corwin et al., 2021), and regular staff-patient contact (Lausch et al., 2003) reportedly increased trust in services. Navigation of care systems was facilitated through simple pathways (Latoo et al., 2021) or support with follow-ups and referrals (Lausch et al., 2003; Luque et al., 2012), including in the next destination of the mobile workers (Garcia et al., 2012; Gruchy and Kapilashrami, 2019; Price et al., 2013).

To increase service *acceptability* for migrant workers, linguistic and cultural differences were addressed, by engaging multilingual staff (Corwin et al., 2021; Latoo et al., 2021; Lausch et al., 2003; Lukes and Simon, 2006), translators (Burgel et al., 2004; Connor et al., 2010), CHWs (Ingram et al., 2015), and cultural mediators (Di Gennaro et al., 2021), or by applying digital tools (Liem et al., 2022; Price et al., 2013) and incorporating patients’ health beliefs and practices (Connor et al., 2010; Lausch et al., 2003). Service acceptability reportedly further increased through telehealth services (making uptake flexible and independent of employer authorisation (Latoo et al., 2021; Liem et al., 2022)), employer involvement (Brumitt et al., 2011; Chen et al., 2010; Connor et al., 2010; Corwin et al., 2021), and by providing mental health services in a general hospital to reduce stigmatisation (Latoo et al., 2021).

As most targeted services were *available* where migrant workers lived or worked or offered telehealth options (Corwin et al., 2021; Latoo et al., 2021; Liem et al., 2022), transport-related barriers (including time and cost) were often circumvented. Clinic times were sometimes harmonised with patients’ working hours by offering weekend or evening services (Burgel et al., 2004; Connor et al., 2010; Corwin et al., 2021; Lukes and Simon, 2006; Luque et al., 2012). While service availability was overall improved, it varied considerably, from the around-the-clock opening of the Qatari hospital (Latoo et al., 2021) to irregular and intermittent mobile clinic visits and the differing compatibility of clinic and working hours (Crouse et al., 2010; Hiebert and Vargas, 2015).

Most healthcare services seemed to be *affordable* through low- or no-cost services, since financial constraints were commonly described as impeding access. If detailed, services were mostly free or highly subsidised (Burgel et al., 2004; Chen et al., 2010; Connor et al., 2007; Di Gennaro et al., 2021; Etienne et al., 2016; Gruchy and Kapilashrami, 2019; Heravi and Bertram, 2007; Hiebert and Vargas, 2015; Latoo et al., 2021) and accessible independent of insurance status (Corwin et al., 2021).

To ensure *appropriate* type and quality of services, qualified healthcare staff, including with specialisations in mental and occupational health (Burgel et al., 2004; Chen et al., 2010; Garcia et al., 2012; Heravi and Bertram, 2007; Latoo et al., 2021) were engaged. Sometimes not all necessary medical specialties were available (Crouse et al., 2010; Etienne et al., 2016), due to which referrals to other services were made (Brumitt et al., 2011; Burgel et al., 2004; Lausch et al., 2003; Luque et al., 2012). However, other transport- and cost-related barriers could continue to impede access into wider care structures (Burgel et al., 2004). In some cases, migrant workers’ living situation (Connor et al., 2010) and mobility were considered when providing and planning treatments: Continuity of care for mobile workers was sought through telehealth interventions (Liem et al., 2022; Price et al., 2013), virtual care management in the USA (offering navigation support, transfer of medical records, and referrals) (Garcia et al., 2012), and patient-held medical records, higher medication supplies, and transfer letters in South Africa (Gruchy and Kapilashrami, 2019).

### 3.4 Effects of healthcare services

The subset of six studies with widely ranging participant numbers and mixed quality provide scattered evidence on the effects of the healthcare services (Table 2).

**Table 2:** Overview of studies evaluating healthcare service effects (N = 6).

Study	Country, population	Intervention	Study design, *outcomes addressed*	Methods, number of participants	Results	Quality
Chen et al. (2010)	China, factory workers (mostly internal migrants)	Occupational health services	Quasi-experimental study (pre- post assessment) between 2006 and 2008 *Health outcomes (knowledge), access*	Quantitative survey of occupational health-related knowledge (150 managers and 4,500 workers in each year); comparison of service coverage	• Increased occupational health knowledge (from 66/150 to 143/150 managers and from 1,347/4,500 to 4,043/4,500 workers) • Increased coverage of occupational health services of factories (35 %–82 %) and workers (29 %–81 %)	Low
Lukes and Simon (2006)	USA, migrant and seasonal farmworkers	Dental services in federally-funded health centres	Cross-sectional descriptive study *Service use, access*	Quantitative survey of health centres (N = 81; 41 % response)	• Proportion of service use: emergency (44 %), basic restorative (32 %), preventive (26 %) care • Rating of perceived access barriers: cost, transportation, knowledge about services, opening times, fear of dental work, language	Medium
Hiebert and Vargas (2015)	Dominican Republic, migrant farmworkers	Mobile clinic	Cross-sectional study *Service use, satisfaction*	Survey on utilisation and perception of services (N = 173)	• 58 % had visited a mobile clinic • Most users are female (75 %) • 92 % described quality of mobile clinic as good or very good • 76 % rated the quality of mobile clinics as better than local clinics • 88 % always trust foreign doctors of mobile clinics	High
Crouse et al. (2010)	Dominican Republic, migrant workers (mostly agriculture)	Mobile clinic	Mixed-methods study^a^ Access	Qualitative interviews with healthcare staff and patients (N = 30)	• Mobile services only provide intermittent care • Lack of emergency care access • Need for standardised referral system	Low
Liem et al. (2022)	China, Filipino migrant workers (mostly domestic work)	Mental health app	Mixed-methods study^a^ *Health outcomes, accessibility, acceptability*	Qualitative interviews with app users (N = 25)	• Improved understanding of and coping with mental health problems (e.g. relaxation, improved social support) • Technical accessibility was an issue for some • App accepted by users	High
Price et al. (2013)	USA, migrant farmworkers	App for managing chronic diseases	Cross-sectional study *Access, acceptability*	Quantitative survey of potential app users (N = 80)	• 81 % mobile phone ownership • 81 % would likely or definitely use the app and perceived it as useful	Low

a Only the qualitative part was critically appraised and included in the synthesis.

Healthcare access and uptake were examined in different ways. The coverage of the occupational health system in Bao'an increased from 610,000 to 1.9 million workers and from 35 % to 82 % of factories between 2006 and 2008 (Chen et al., 2010). Lukes and Simon surveyed health centres across the USA providing dental services to migrant farmworkers. Service use was dominated by emergency care (44 %), while restorative (32 %) and preventative (26 %) services accounted for fewer visits than aspired, indicating delayed care seeking. Surveyed health centre representatives ranked cost and transport (same ranking), insufficient knowledge of services, limited clinic hours, and language barriers as the most common access barriers (Lukes and Simon, 2006). Hiebert and Vargas (2014) found that young adults and males in agricultural communities in the Dominican Republic visited mobile clinics less frequently than women and older people, which raised the question regarding potential differences in the delivery of services for different groups and service acceptability. Based on qualitative interviews with farmworkers and healthcare providers in the Dominican Republic, Crouse et al. (2010) reported lacking emergency care access between periodic mobile clinic visits and the need for a standardised referral system for higher level care. The two telehealth studies evaluated accessibility in terms of mobile phone ownership, which was found to be high (81 % in 2011–2012) among migrant farmworkers in the USA (Price et al., 2013), and technical accessibility while using the mental health app, where Filipino migrant workers encountered different technical challenges (Liem et al., 2022).

Two studies examined health-related outcomes, pointing to improved mental health literacy and well-being through a mental health app for Filipino migrant workers (based on qualitative interviews) (Liem et al., 2022) and increased occupational health-related knowledge of Chinese factory employers after two years of occupational health services and training (based on survey data) (Chen et al., 2010).

Three studies evaluated patient satisfaction and acceptability, indicating positive effects. Surveys yielded superior patient assessments of mobile clinics compared to local services in the Dominican Republic (Hiebert and Vargas, 2015) and high levels of willingness to use the app for chronic disease management among farmworkers in the USA reported they would likely or definitely use (Price et al., 2013). Liem et al. (2022) concluded from qualitative interviews that the mental health app for overseas Filipino workers was well-accepted.

## 4 Discussion

Migrant workers comprise one of the most important cohorts in the world's basic production and service sectors. They are also often the individuals who are exposed to the greatest health risks and most substantial barriers to healthcare based on multidimensional othering and systemic bordering of labour and social protections and health services. This review identified 21 health-related healthcare services for migrant workers in six countries that attempt to overcome systemic borders. These services included diverse models of care, including mobile clinics, established healthcare facilities and telehealth interventions provided by governmental, NGO, academic, and private actors. Ultimately, however, most documented services targeted farmworkers and were based in the USA, while none were identified in low and lower-middle income countries.

### 4.1 Healthcare service effects

The extent to which the healthcare services influenced workers’ access and uptake, their health, patient satisfaction, and acceptability or were cost-effective remains largely unknown due to limited evaluative evidence. This finding echoes previous remarks about the need for more intervention research and evaluations on health services for low-wage and migrant workers (Abubakar et al., 2018; Arcury and Quandt, 2007; Hiebert and Vargas, 2015; Luque and Castañeda, 2013), and mobile clinics, in general (Beks et al., 2020; McGowan et al., 2020). However, the absence of evaluations might also be an artefact of the database-focussed search strategy, since programme evaluations are not always published in academic forums (Beks et al., 2020; Luque and Castañeda, 2013).

### 4.2 Healthcare access

The healthcare services adopted various strategies to address commonly reported access barriers that exclude low-wage and migrant workers from health systems. Results indicate that financial barriers were overcome almost universally through low-cost or free care. While the availability of healthcare services was generally improved, findings also indicate remaining service gaps due to intermittent outreach visits or the incompatibility of service times with patients’ working hours. Furthermore, mobile or smaller place-based clinics only offered a limited range of services, often contingent on individual staff members, which potentially decreases the appropriateness of care, i.e., the fit between services and patient needs (Levesque et al., 2013). Many publications mentioned that interventions addressed linguistic and cultural differences. However, it often remained unclear how (and whether) this was achieved and perceived by patients or whether othering was (unintentionally) reinforced by reproducing specific social categories. Truly non-discriminatory and patient-centred care avoids cultural essentialism and the reduction of “culture” to language. Healthcare services for indigenous communities, often based on community engagement, offer valuable examples for effectively making services culturally-sensitive (Harfield et al., 2018). Services were also made more appropriate for mobile workers by assuring continuity of care, e.g., through transferred or patient-held medical records. The US Health Network, a virtual case management with links to 120 countries (Migrant Clinician Network, 2024), exemplifies cross-border care that benefits mobile patients as a whole. While the different ways in which the reviewed interventions facilitated healthcare access may offer valuable examples for overcoming the multidimensional access barriers commonly faced by low-wage migrant workers, the overall accessibility of services remains unclear.

Based on the review findings, the potential of telehealth for this mobile population seems to be relatively untapped. Telehealth services generally show high effectiveness (Snoswell et al., 2021), which can lower access barriers related to service gaps, transport, language, and time, and decrease dependency on employer consent for care seeking. However, telehealth can also reconfigure barriers (Hynie et al., 2022). For example, the Non-Resident Nepali Association organised multidisciplinary telemedicine services during the COVID-19 pandemic to connect Nepalis based abroad with health professionals through various digital technologies (e.g., email, telephone, video calls). Insufficient transborder regulations for providing medical consultations and prescriptions, digital gaps, and low literacy levels of some patients posed challenges (Sapkota et al., 2022). Technology and literacy barriers have also been reported for other populations using telehealth services, e.g., racial and ethnic minority groups (Hynie et al., 2022; Truong et al., 2022). Telehealth interventions may thus also increase inequities in access for migrant workers and therefore need careful planning.

Engagement of patient and community members figured across the five access dimensions in this review. In particular, CHWs linked patients and services, making services more approachable through information and trust-building, lowering linguistic and cultural barriers, and improving availability through basic healthcare. A recent review by the World Health Organisation concluded that CHWs have “enormous potential to extend health care services to vulnerable populations”, including through curative services (World Health Organization, 2020). The sustainability and effectiveness of CHW programmes was improved by their embeddedness in national health systems and communities as well as appropriate training and support of CHWs (World Health Organization, 2021).

### 4.3 Integration with wider health systems

This review raises the question of service integration into wider care structures, which varied across the reviewed healthcare services. While some of the reviewed services, such as the Qatari industrial hospital, the occupational health system in Bao'an and the country-wide network of migrant health clinics in the USA, were clearly linked to a broader healthcare system, formalised links to other healthcare services were lacking for some of the identified mobile clinics. Furthermore, in some cases where efforts were made to generate links to the broader healthcare system, barriers (e.g., related to transport and costs) persisted, making referral systems dysfunctional.

For healthcare to be appropriate, services must meet needs (Levesque et al., 2013), which requires referral options for more complex needs, as stressed by the International Committee of the Red Cross mobile clinic directives (International Commitee of the Red Cross, 2006). This poses particular challenges where health systems are overburdened. Non-governmental and private corporate activities can fill resulting service gaps but are often of limited sustainability and scope and may trigger service fragmentation (Gruchy and Kapilashrami, 2019; Pfeiffer et al., 2008; Sharma, 2014). Thus, a reliance on non-governmental actors can undermine overall health system strengthening (Pfeiffer et al., 2008). Furthermore, employer-provided healthcare might be unacceptable for workers who fear negative repercussions from disclosing ill-health (Baron et al., 2014).

Ultimately, national governments are responsible for population health (Office of United Nations High Commissioner for Human Rights & World Health Organization, 2008) but these are also the same entities that intentionally or neglectfully structure health systems that exclude or omit migrants. Indeed, a recent UK study on healthcare and education structures highlighted the securitisation of these basic services by requiring data-sharing to advance the UK Home Office immigration agenda (Cassidy and Davidson, 2024). Similarly, securitisation of health in LMICs, such as Malaysia, intensified during the COVID-19 pandemic, deterring undocumented migrants from accessing essential healthcare services, which hindered both preventive and curative efforts (Loganathan et al., 2024).

To achieve advancements towards health equity, health systems must offer diversity-sensitive services that make appropriate efforts to include migrant workers, independent of their immigration status (Abubakar et al., 2018; Luo and Escalante, 2018). Service delivery must consider the multidimensional bordering that excludes workers by integrating healthcare services that are sensitive to diverse needs, especially of full-time workers, into national health systems, while avoiding parallel and unsustainable structures. Diversity-sensitive systems will benefit from a migrant patient-centred understanding of healthcare and access priorities. For instance, undocumented migrants in Italy have legal access to health services (Abubakar et al., 2018), but the reviewed mobile clinic for migrant farmworkers in Italy (Di Gennaro et al., 2021) illustrates that these entitlements cannot be equated with an actual opportunity for access. Therefore, until health systems provide equitable access to this population, targeted interventions have to bridge prevailing gaps but should not function in isolation. In fact, collaborations with other healthcare providers, NGOs, and employers were identified as a major facilitator among the reviewed services. This is congruent with findings from a related review, attributing mobile clinic sustainability to long-term involvement of different organisations, including academic and community partners (Luque and Castañeda, 2013). Thus, vertical approaches specifically targeting the needs of low-wage labour migrants are needed but should converge with horizontal efforts that aim to improve the accessibility of health systems more broadly.

### 4.4 Living and working conditions

While beyond the focus of the present study, it needs to be acknowledged that healthcare is only one, and not necessarily the most impactful, determinant of health on a population level (Frieden, 2010; Solar and Irwin, 2010). Thus, in addition to providing accessible healthcare, other multilevel and multisectoral approaches are needed for improving the health of low-wage workers (Baron et al., 2014; Ingram et al., 2021). Importantly, the living and working conditions, that also influence healthcare needs and the possibilities for healthcare access (Supplement 1), need to be assessed and addressed. Health promotion interventions (Evagora-Campbell et al., 2022; Harris et al., 2014; Pham et al., 2020; Stiehl et al., 2018) may contribute to general health protection, combined with structural level shifts. For example, in line with the International Labour Organisation's Decent Work Agenda (International Labour Organization, n.d.) health promotion for migrant workers would include humane immigration laws, workplace health and safety regulations, paid sick leave, adequate social protection and living wages (Baron et al., 2014; Ingram et al., 2021). A multicountry case-study on the meat industry during the Covid-19 pandemic illustrates how national governance approaches can differ from industry support to systemic change towards more equitable policies (Gottlieb et al., 2025). Proactive policy actions on living and working conditions can lead to measurable health improvements, e.g., findings from a natural experiment indicate that the introduction of minimum wages in the United Kingdom in 1999 significantly improved low-wage workers' mental health (Reeves et al., 2017).

### 4.5 Limitations

When interpreting these results, the limitations and characteristics of the identified body of evidence have to be considered. There was a strong focus on the agricultural sector, migrant workers, and, geographically, the USA – reflecting bibliometric findings (Sweileh, 2018). Included titles contained varying levels of relevant information, which was mostly descriptive. Theoretical underpinnings of the interventions were overall lacking. The sparse evaluative evidence was mostly of limited quality.

The perspective offered by the present review is limited by its methodological approach, including the language constraints and the briefness of the grey literature search. Furthermore, while the search terms for the work sectors were selected carefully, they do not cover the entire global population of low-wage labour migrants, who are engaged in a wide range of activities. Relevant interventions offering valuable insights might also have been missed by excluding services also targeting other patient groups or interventions that aim to facilitate access to wider care structures.

### 4.6 Implications

This review has important implications for overcoming othering and healthcare bordering practices, and improving policies for inclusive systems. For healthcare decision-makers, findings indicate common access barriers and forms of exclusion, and suggest strategies designed to respond to migrant workers’ needs. The strategies outlined in Table 1 can inspire changes in health systems, but these should be implemented with great care given contextual differences between health systems and the very limited evaluative evidence. Achieving inclusive healthcare and move towards greater health equity in responding to social determinants of health will also benefit from multisectoral collaborations, e.g., between healthcare providers, governmental agencies, employers, and, importantly, the patient community.

Budget allocations are central to making health systems accessible and effective for migrant workers and similarly excluded population groups. Moreover, the medical profession will benefit from including training on migrant-inclusive services into medical curricula and clinical practice. For example, occupational safety and health, plus diversity-sensitive service essentials should form part of medical school curricula and migrant-aware clinical intake processes (Simmons et al., 2018). Moreover, inclusive health strategies will subsidise care for uninsured and undocumented patients (Luo and Escalante, 2018; Simmons et al., 2018), including adequate insurance coverage and making health systems more “migration-aware” (Vearey et al., 2017). Internationally, regulations for cross-border healthcare provision, including prescriptions, need to be further established, perhaps by exploring the potential of telehealth strategies (Sapkota et al., 2022). As noted, healthcare policies cannot be undermined by exclusionary labour, immigration, and social policies.

Apart from the general need for more research on low-wage and migrant workers' health (Sweileh, 2018), more studies on targeted interventions are needed – preferably with longitudinal mixed-method designs capturing longer-term effects, including on equity in access, cost-effectiveness, programme sustainability, and patient perspectives. In particular, the potential of telehealth services for this mobile population should be further examined. In parallel, more extensive reviews of literature available outside of academic forums should be undertaken and past evaluations should be made more widely available. In addition to targeted interventions, the research focus should also be directed to measures aiming to facilitate migrant workers’ access into the wider health system (e.g., through CHWs or insurance schemes).

## 5 Conclusion

Low-wage migrant workers are a heterogeneous population who sustain numerous crucial labour sectors, yet they often encounter multiple health risks and exclusion from healthcare. Given the global prevalence of labour migration (International Labour Organization, 2021; McAuliffe and Triandafyllidou, 2021), health equity via universal health coverage can only be achieved if we meet the healthcare needs of migrant workers. Healthcare, while integral, can only be part of a strategy to protect the health of these *not* invisible but often overlooked workers.

**Table:** Abbreviations

CHW	Community health worker
COVID-19	Corona virus disease of 2019
ICRC	International Committee of the Red Cross
LMICs	Low- and middle-income countries
LSHTM	London School of Hygiene & Tropical Medicine
JBI	Joanna Briggs Institute
NGO	non-governmental organisation
PHC	Primary healthcare
PICO	Population, Intervention, Comparator, Outcome
PRISMA	Preferred Reporting Items for Systematic Reviews
PROSPERO	International Prospective Register of Systematic Reviews

## CRediT authorship contribution statement

**Eilin Rast:** Writing – review & editing, Writing – original draft, Validation, Project administration, Methodology, Investigation, Formal analysis, Data curation, Conceptualization. **Karen Lau:** Writing – review & editing, Validation, Methodology. **Rosita Chia-Yin Lin:** Writing – review & editing, Validation. **Tharani Loganathan:** Writing – review & editing, Validation. **Sally Hargreaves:** Writing – review & editing, Supervision, Project administration, Methodology. **Cathy Zimmerman:** Writing – review & editing, Validation, Supervision, Project administration, Methodology, Conceptualization.

## Ethics approval and consent to participate

Not applicable.

## Consent for publication

Not applicable.

## Availability of data and materials

This review is based on published literature only.

## Ethics declaration

No ethical approval was needed for the conduct of this literature review.

## Funding

ER was supported by a fellowship of the 10.13039/100021828German Academic Exchange Service (DAAD). KL was supported by an 10.13039/501100000265MRC grant (MR/W006677/1). RCL was supported by a scholarship from the Ministry of Education in Taiwan. Funders had no role in the conceptualisation, design, data collection, analysis, decision to publish, or preparation of the manuscript.

## Declaration of competing interests

All authors declare no declarations of interest.

## Data Availability

No data was used for the research described in the article.

## References

[bib1] Abad N., Baack B.N., O'Leary A., Mizuno Y., Herbst J.H., Lyles C.M. (2015). A systematic review of HIV and STI behavior change interventions for female sex workers in the United States. AIDS Behav..

[bib2] Abubakar I., Aldridge R.W., Devakumar D., Orcutt M., Burns R., Barreto M.L., Dhavan P., Fouad F.M., Groce N., Guo Y., Hargreaves S., Knipper M., Miranda J.J., Madise N., Kumar B., Mosca D., McGovern T., Rubenstein L., Sammonds P., Zimmerman C. (2018). The UCL-Lancet Commission on Migration and Health: the health of a world on the move. Lancet (London, England).

[bib3] Akbulut N., Razum O. (2022). Why Othering should be considered in research on health inequalities: theoretical perspectives and research needs. SSM - Population Health.

[bib4] Aktas E., Bergbom B., Godderis L., Kreshpaj B., Marinov M., Mates D., McElvenny D.M., Mehlum I.S., Milenkova V., Nena E., Glass D.C. (2022). Migrant workers occupational health research: an OMEGA-NET working group position paper. Int. Arch. Occup. Environ. Health.

[bib5] Andersen R.M. (1995). Revisiting the behavioral model and access to medical care: does it matter?. J. Health Soc. Behav..

[bib6] Arcury T.A., Quandt S.A. (2007). Delivery of health services to migrant and seasonal farmworkers. Annu. Rev. Publ. Health.

[bib7] Baron S.L., Beard S., Davis L.K., Delp L., Forst L., Kidd-Taylor A., Liebman A.K., Linnan L., Punnett L., Welch L.S. (2014). Promoting integrated approaches to reducing health inequities among low-income workers: applying a social ecological framework. Am. J. Ind. Med..

[bib8] Beks H., Ewing G., Charles J.A., Mitchell F., Paradies Y., Clark R.A., Versace V.L. (2020). Mobile primary health care clinics for Indigenous populations in Australia, Canada, New Zealand and the United States: a systematic scoping review. Int. J. Equity Health.

[bib9] Bloss J.E., LePrevost C.E., Zahra A.G., Firnhaber G.C., Cofie L.E., Zepeda R., Lee J.G.L. (2022). Advancing the health of migrant and seasonal farmworkers in the United States: identifying gaps in the existing literature, 2021. Health Promot. Pract..

[bib10] Brumitt J., Garside L.I., Reisch R., Marshall T., Gilpin H.E., Kinsey J., Imondi K. (2011). Musculoskeletal healthcare for latino migrant farmworkers: interprofessional collaboration to provide service and educate future healthcare providers. Health and Interprofessional Practice.

[bib11] Buller A.M., Stoklosa H., Zimmerman C. (2015).

[bib12] Burgel B.J., Lashuay N., Israel L., Harrison R. (2004). Garment workers in California: health outcomes of the asian immigrant women workers clinic. AAOHN J. : Official Journal of the American Association of Occupational Health Nurses.

[bib13] Cassidy K., Davidson G. (2024). Bordering public institutions through the routinization of borderwork and datafication: internalized immigration regimes within UK health care and higher education. Environ. Plann. Soc. Space.

[bib14] Chen Y., Chen J., Sun Y., Liu Y., Wu L., Wang Y., Yu S. (2010). Basic occupational health services in Baoan, China. J. Occup. Health.

[bib15] Connor A., Layne L., Thomisee K. (2010). Providing care for migrant farm worker families in their unique sociocultural context and environment. J. Transcult. Nurs. : Official Journal of the Transcultural Nursing Society.

[bib16] Connor A., Rainer L.P., Simcox J.B., Thomisee K. (2007). Increasing the delivery of health care services to migrant farm worker families through a community partnership model. Public Health Nurs..

[bib17] Corwin C., Sinnwell E., Culp K. (2021). A mobile primary care clinic mitigates an early COVID-19 outbreak among migrant farmworkers in Iowa. J. Agromed..

[bib18] Crouse H.L., Macias C.G., Cruz A.T., Wilson K.A., Torrey S.B. (2010). Utilization of a mobile medical van for delivering pediatric care in the bateys of the Dominican Republic. Int. J. Emerg. Med..

[bib19] Dhana A., Luchters S., Moore L., Lafot Y., Roy A., Scorgie F., Chersich M. (2014). Systematic review of facility-based sexual and reproductive health services for female sex workers in Africa. Glob. Health.

[bib20] Di Gennaro F., Lattanzio R., Falanga C., Negri S., Papagni R., Novara R., Panico G.G., Totaro V., Poliseno M., Bavaro D.F., Raho L., Schiavone M., Laforgia N., Volpe A., Laforgia R., Lo Caputo S., Marotta C., Putoto G., Saracino A. (2021). Low-wage agricultural migrant workers in apulian ghettos, Italy: general health conditions assessment and HIV screening. Trop. Med. Infect. Dis..

[bib21] Etienne M.O., Messmer P.R., Danis S.J., Blot G. (2016). Outcomes of an immersion proiect in the Dominican republic bateyes. J. Natl. Black Nurses' Assoc. JNBNA : J. Natl. Black Nurses' Assoc. JNBNA.

[bib22] European Observatory of Working Life (2018). *Precarious work.* European foundation for the improvement of living and working conditions. https://www.eurofound.europa.eu/observatories/eurwork/industrial-relations-dictionary/precarious-work.

[bib23] Evagora-Campbell M., Zahidie A., Buse K., Rabbani F., Hawkes S. (2022). Promoting labour migrant health equity through action on the structural determinants: a systematic review. Journal of Migration and Health.

[bib24] Falconer J. (2018). Removing duplicates from an EndNote library.

[bib25] Flynn M.B. (2021). Global capitalism as a societal determinant of health: a conceptual framework. Soc. Sci. Med..

[bib26] Frieden T.R. (2010). A framework for public health action: the health impact pyramid. Am. J. Publ. Health.

[bib27] Garcia D., Hopewell J., Liebman A.K., Mountain K. (2012). The migrant clinicians network: connecting practice to need and patients to care. J. Agromed..

[bib28] Goldman S., Aspenson Anna, Bhatnagar Prashasti, Martin R. (2021).

[bib29] Gottlieb N., Jungwirth I., Glassner M., Lange T. de, Mantu S., Forst L. (2025). Immigrant workers in the meat industry during COVID-19: comparing governmental protection in Germany, The Netherlands, and the USA. Glob. Health.

[bib30] Grimshaw D. (2011). http://www.ilo.org/wcmsp5/groups/public/---ed_protect/---protrav/---travail/documents/publication/wcms_157253.pdf.

[bib31] Grove N.J., Zwi A.B. (2006). Our health and theirs: forced migration, othering, and public health. Soc. Sci. Med..

[bib32] Gruchy T. de, Kapilashrami A. (2019). After the handover: exploring MSF's role in the provision of health care to migrant farm workers in Musina, South Africa. Glob. Public Health.

[bib33] Harfield S.G., Davy C., McArthur A., Munn Z., Brown A., Brown N. (2018). Characteristics of Indigenous primary health care service delivery models: a systematic scoping review. Glob. Health.

[bib34] Hargreaves S., Rustage K., Nellums L.B., McAlpine A., Pocock N., Devakumar D., Aldridge R.W., Abubakar I., Kristensen K.L., Himmels J.W., Friedland J.S., Zimmerman C. (2019). Occupational health outcomes among international migrant workers: a systematic review and meta-analysis. Lancet Global Health.

[bib35] Harris J.R., Hannon P.A., Beresford S.A.A., Linnan L.A., McLellan D.L. (2014). Health promotion in smaller workplaces in the United States. Annu. Rev. Publ. Health.

[bib36] Heravi M., Bertram J.E.A. (2007). A novel resource model for underprivileged health support: community Medical Outreach. Rural Remote Health.

[bib77] Hiebert L., Vargas G. (2015). Utilization Patterns and Perceptions of Mobile Helath Clinics in Batey Libertad, Dominican Republic. The Columbia University Journal of Global Health..

[bib37] Hynie M., Jaimes A., Oda A., Rivest-Beauregard M., Perez Gonzalez L., Ives N., Ahmad F., Kuo B.C.H., Arya N., Bokore N., McKenzie K. (2022). Assessing virtual mental health access for refugees during the COVID-19 pandemic using the levesque client-centered framework: what have we learned and how will we plan for the future?. Int. J. Environ. Res. Publ. Health.

[bib38] Ingram M., Schachter K.A., Guernsey de Zapien J., Herman P.M., Carvajal S.C. (2015). Using participatory methods to enhance patient-centred mental health care in a federally qualified community health center serving a Mexican American farmworker community. Health Expect. : An International Journal of Public Participation in Health Care and Health Policy.

[bib39] Ingram M., Wolf A.M.A., López-Gálvez N.I., Griffin S.C., Beamer P.I. (2021). Proposing a social ecological approach to address disparities in occupational exposures and health for low-wage and minority workers employed in small businesses. J. Expo. Sci. Environ. Epidemiol..

[bib40] Innes A. (2024). International Politics.

[bib41] International Commitee of the Red Cross (2006).

[bib42] International Labour Office (2017).

[bib43] International Labour Organization. (n.d.). Decent work. Retrieved August 27, 2023, from https://www.ilo.org/global/topics/decent-work/lang--en/index.htm.

[bib44] International Labour Organization (2010).

[bib45] International Labour Organization (2021).

[bib46] International Labour Organization, Walk Free, & International Organization for Migration (2022).

[bib47] Jeal N., Macleod J., Turner K., Salisbury C. (2015). Systematic review of interventions to reduce illicit drug use in female drug-dependent street sex workers. BMJ Open.

[bib48] Joanna Briggs Institute. Critical Appraisal Tools. https://jbi.global/critical-appraisal-tools.

[bib49] Johnson L., Potter L.C., Beeching H., Bradbury M., Matos B., Sumner G., Wills L., Worthing K., Aldridge R.W., Feder G., Hayward A.C., Pathak N., Platt L., Story A., Sultan B., Luchenski S.A. (2023). Interventions to improve health and the determinants of health among sex workers in high-income countries: a systematic review. Lancet Public Health.

[bib50] Krieger N. (2010). Workers are people too: societal aspects of occupational health disparities--an ecosocial perspective. Am. J. Ind. Med..

[bib51] Ladegaard H.J. (2022). Language, discrimination and employability: employers' othering and racist representations of domestic migrant workers on social media. J. Lang. Soc. Psychol..

[bib52] Latoo J., Wadoo O., Iqbal Y., Chandrappa N.S.K., Tulley I., Alabdulla M. (2021). Development of mental health services for lower-skilled migrant workers in Qatar. Asian Journal of Psychiatry.

[bib53] Lausch C., Heuer L., Guasasco C., Bengiamin M. (2003). The experiences of migrant health nurses employed in seasonal satellite nurse-managed centers: a qualitative study. J. Community Health Nurs..

[bib54] Levesque J.-F., Harris M.F., Russell G. (2013). Patient-centred access to health care: conceptualising access at the interface of health systems and populations. Int. J. Equity Health.

[bib55] Liem A., Pakingan K.A., Garabiles M.R., Sit H.F., Burchert S., Lam A.I.F., Hall B.J. (2022). Evaluating the implementation of a mental health app for overseas Filipino workers in Macao China: a mixed-methods study of stakeholders' perspectives. Front. Psychiatr..

[bib56] Loganathan T., Chan Z.X., Pocock N.S. (2020). Healthcare financing and social protection policies for migrant workers in Malaysia. PLoS One.

[bib57] Loganathan T., Rui D., Ng C.-W., Pocock N.S. (2019). Breaking down the barriers: understanding migrant workers’ access to healthcare in Malaysia. PLoS One.

[bib58] Loganathan T., Zaini A.Z., Kunpeuk W., Suphanchaimat R., Yi H., Farwin A., Abdul Majid H. (2024). Challenges faced by migrant populations in complying with public health measures during the COVID-19 pandemic in Malaysia: a qualitative study. BMJ Public Health.

[bib59] Lukes S.M., Simon B. (2006). Dental services for migrant and seasonal farmworkers in US community/migrant health centers. J. Rural Health : Official Journal of the American Rural Health Association and the National Rural Health Care Association.

[bib60] Luo T., Escalante C.L. (2018). Health care service utilization of documented and undocumented hired farmworkers in the U.S. Eur. J. Health Econ. : HEPAC : Health Economics in Prevention and Care.

[bib61] Luque J.S., Castañeda H. (2013). Delivery of mobile clinic services to migrant and seasonal farmworkers: a review of practice models for community-academic partnerships. J. Community Health.

[bib62] Luque J.S., Reyes-Ortiz C., Marella P., Bowers A., Panchal V., Anderson L., Charles S. (2012). Mobile farm clinic outreach to address health conditions among Latino migrant farmworkers in Georgia. J. Agromed..

[bib63] Marcin J.P., Shaikh U., Steinhorn R.H. (2016). Addressing health disparities in rural communities using telehealth. Pediatr. Res..

[bib64] Marmot M. (2016).

[bib65] McAuliffe M., Triandafyllidou A. (2021).

[bib66] McGowan C.R., Baxter L., Deola C., Gayford M., Marston C., Cummings R., Checchi F. (2020). Mobile clinics in humanitarian emergencies: a systematic review. Conflict Health.

[bib67] McKenzie J.E., Brennan S.E., Ryan R.E., Thomson H.J., Johnston R.V., Thomas J., Higgins J., Thomas J., Chandler J., Cumpston M., Li T., Page M., Welch V. (2023).

[bib69] Mucci N., Traversini V., Giorgi G., Garzaro G., Fiz-Perez J., Campagna M., Rapisarda V., Tommasi E., Montalti M., Arcangeli G. (2019). Migrant workers and physical health: an umbrella review. Sustainability.

[bib70] O'Donnell P., O'Donovan D., Elmusharaf K. (2018). Measuring social exclusion in healthcare settings: a scoping review. Int. J. Equity Health.

[bib71] Office of United Nations High Commissioner for Human Rights, & World Health Organization (2008).

[bib72] Ottisova L., Hemmings S., Howard L.M., Zimmerman C., Oram S. (2016). Prevalence and risk of violence and the mental, physical and sexual health problems associated with human trafficking: an updated systematic review. Epidemiol. Psychiatr. Sci..

[bib73] Ouzzani M., Hammady H., Fedorowicz Z., Elmagarmid A. (2016). Rayyan-a web and mobile app for systematic reviews. Syst. Rev..

[bib74] Page M.J., McKenzie J.E., Bossuyt P.M., Boutron I., Hoffmann T.C., Mulrow C.D., Shamseer L., Tetzlaff J.M., Akl E.A., Brennan S.E., Chou R., Glanville J., Grimshaw J.M., Hróbjartsson A., Lalu M.M., Li T., Loder E.W., Mayo-Wilson E., McDonald S., Moher D. (2021). The PRISMA 2020 statement: an updated guideline for reporting systematic reviews. BMJ, Br. Med. J. (Clin. Res. Ed.).

[bib75] Parikh K., Marein-Efron G., Huang S., O'Hare G., Finalle R., Shah S.S. (2010). Nutritional status of children after a food-supplementation program integrated with routine health care through mobile clinics in migrant communities in the Dominican Republic. Am. J. Trop. Med. Hyg..

[bib76] Pega F., Govindaraj S., Tran N.T. (2021). Health service use and health outcomes among international migrant workers compared with non-migrant workers: a systematic review and meta-analysis. PLoS One.

[bib78] Pfeiffer J., Johnson W., Fort M., Shakow A., Hagopian A., Gloyd S., Gimbel-Sherr K. (2008). Strengthening health systems in poor countries: a code of conduct for nongovernmental organizations. Am. J. Publ. Health.

[bib79] Pham C.T., Phung D., Nguyen T.V., Chu C. (2020). The effectiveness of workplace health promotion in low- and middle-income countries. Health Promot. Int..

[bib80] Pocock N.S., Nguyen L.H., Lucero-Prisno Iii D.E., Zimmerman C., Oram S. (2018). Occupational, physical, sexual and mental health and violence among migrant and trafficked commercial Fishers and seafarers from the Greater Mekong Subregion (GMS): systematic review. Global Health Research and Policy.

[bib81] Price M., Williamson D., McCandless R., Mueller M., Gregoski M., Brunner-Jackson B., Treiber E., Davidson L., Treiber F. (2013). Hispanic migrant farm workers' attitudes toward mobile phone-based telehealth for management of chronic health conditions. J. Med. Internet Res..

[bib82] Qian X., Smith H., Huang W., Zhang J., Huang Y., Garner P. (2007). Promoting contraceptive use among unmarried female migrants in one factory in Shanghai: A pilot workplace intervention. BMC Health Serv. Res..

[bib83] Rast E., Lau K., Lin R.C.-Y., Hargreaves S., Zimmerman C. (2023). Healthcare interventions targeting low-wage workers across the globe: A systematic review. PROSPERO 2023 CRD42023459360.

[bib84] Reeves A., McKee M., Mackenbach J., Whitehead M., Stuckler D. (2017). Introduction of a national minimum wage reduced depressive symptoms in low-wage workers: a quasi-natural experiment in the UK. Health Econ..

[bib85] Rinaldi G., Kiadaliri A.A., Haghparast-Bidgoli H. (2018). Cost effectiveness of HIV and sexual reproductive health interventions targeting sex workers: a systematic review. Cost Eff. Resour. Allocation : C/E.

[bib86] Saldaña-Villanueva K., Méndez-Rodríguez K.B., Zamora-Mendoza B.N., Gómez-Gómez A., Díaz-Barriga F., Pérez-Vázquez F.J. (2023). Health effects of informal precarious workers in occupational environments with high exposure to pollutants. Environ. Sci. Pollut. Res. Int..

[bib87] Santalahti M., Sumit K., Perkiö M. (2020). Barriers to accessing health care services: a qualitative study of migrant construction workers in a southwestern Indian city. BMC Health Serv. Res..

[bib88] Sapkota S., Adhikari P., Sah S., Bhattarai S., Shrestha S.P., Poudel S., Sharma H.R., Maleku K., Simkhada P. (2022). Use of telehealth services among Nepali living overseas during Covid-19 pandemic: the opportunities, limitations, lessons learned and recommendations. J. Oral Biol. Craniofac. Res..

[bib89] Sharma S. (2014). Mobile health services through CSR initiatives. Journal of Development Management and Communication.

[bib90] Simmons J.M., Liebman A.K., Sokas R.K. (2018). Occupational health in community health centers: practitioner challenges and recommendations. New Solut. : A Journal of Environmental and Occupational Health Policy : NS.

[bib91] Simon J., Kiss N., Łaszewska A., Mayer S. (2015).

[bib92] Snoswell C.L., Chelberg G., Guzman K. R. de, Haydon H.H., Thomas E.E., Caffery L.J., Smith A.C. (2021). The clinical effectiveness of telehealth: a systematic review of meta-analyses from 2010 to 2019. J. Telemed. Telecare.

[bib93] Solar O., Irwin A. (2010).

[bib94] Stiehl E., Shivaprakash N., Thatcher E., Ornelas I.J., Kneipp S., Baron S.L., Muramatsu N. (2018). Worksite health promotion for low-wage workers: a scoping literature review. Am. J. Health Promot. : AJHP.

[bib95] Sweileh W.M. (2018). Global output of research on the health of international migrant workers from 2000 to 2017. Glob. Health.

[bib96] Truong M., Yeganeh L., Cook O., Crawford K., Wong P., Allen J. (2022). Using telehealth consultations for healthcare provision to patients from non-Indigenous racial/ethnic minorities: a systematic review. J. Am. Med. Inf. Assoc. : JAMIA.

[bib97] Turner K., Meyrick J., Miller D., Stopgate L. (2022). Which psychosocial interventions improve sex worker well-being? A systematic review of evidence from resource-rich countries. BMJ Sexual & Reproductive Health.

[bib98] United Nations (2016). Sustainable development goal 3: good health and well-being. https://www.globalgoals.org/goals/3-good-health-and-well-being/.

[bib99] Vearey J., Modisenyane M., Hunter-Adams J. (2017). South African Health Review 2017.

[bib100] Villarejo D. (2003). The health of U.S. Hired farm workers. Annu. Rev. Publ. Health.

[bib101] Vollmer B.A. (2021). Categories, practices and the self – reflections on bordering, ordering and othering. Tijdschr. Econ. Soc. Geogr..

[bib102] World Bank Group (2024). Remittances. https://www.worldbank.org/en/topic/migration/brief/remittances-knomad.

[bib68] Migrant Clinician Network, 2024. Health Network. https://www.migrantclinician.org/our-work/health-network.html.

[bib103] World Health Organization (2008). Closing the Gap in a Generation: Health Equity through Action on the Social Determinants of Health -. Final Report of the Commission on Social Determinants of Health.

[bib104] World Health Organization (2020). What do we know about community health workers? A systematic review of existing reviews. Human Resources for Health Observer Series No 19.

[bib105] World Health Organization (2022).

[bib106] Yuval-Daṿis N., Wemyss G., Cassidy K. (2019). https://ebookcentral.proquest.com/lib/kxp/detail.action?docID=5787866.

[bib107] Zimmerman C., Kiss L. (2017). Human trafficking and exploitation: a global health concern. PLoS Med..

