# Supplementary material for: Healthcare services for low-wage migrant workers: A systematic review
Source: Soc Sci Med. 2025 Sep;380:118176. doi: 10.1016/j.socscimed.2025.118176 (PMC12623714; doi:10.1016/j.socscimed.2025.118176)
Supplement: Multimedia component 1 [file mmc1.docx]

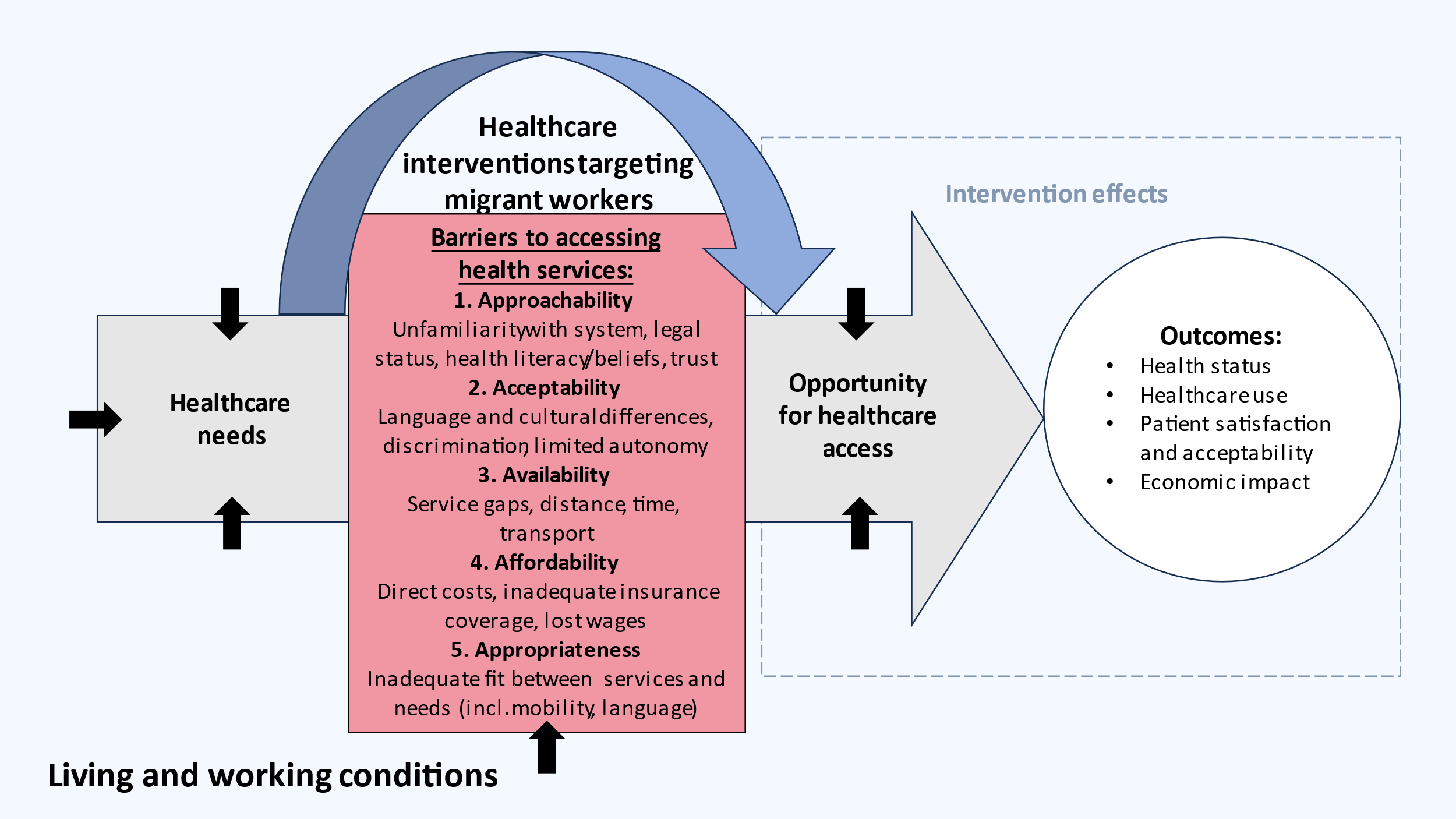


**Supplement 1:** Conceptual framework for the present review of healthcare services for low-wage migrant workers
